# Research Progress and Prospects of Flavonoids in the Treatment of Diseases by Regulating Autophagy: A Narrative Review

**DOI:** 10.3390/molecules31122055

**Published:** 2026-06-11

**Authors:** Shuang Xue, Qiao Wang, Xuan Guo, Xingtong Chen, Yunyue Zhou, Jinbiao Yang, Yukun Zhang, Wenying Niu

**Affiliations:** School of Basic Medical Sciences, Heilongjiang University of Chinese Medicine, Harbin 150040, China

**Keywords:** autophagy, selective autophagy, flavonoids, research progress, disease

## Abstract

Autophagy is an essential mechanism through which cells break down and reuse intracellular proteins and organelles to preserve cellular homeostasis. Under physiological conditions, autophagy primarily exerts a cytoprotective effect; however, aberrant activation or deficiency of autophagy pathways can disturb cellular balance and even trigger apoptosis, thereby contributing to the occurrence and progression of multiple diseases. Flavonoids are natural bioactive components widely distributed in plants, characterized by distinct benefits of synergistic regulation via multiple targets and pathways. This review summarizes the primary mechanisms of flavonoids, focusing on their potential underlying mechanisms against various diseases—including atherosclerosis, cardiovascular diseases, liver diseases, lung diseases, Parkinson’s disease, leukemia, and malignant tumors—via regulating autophagy (including selective autophagy), and sorts out the latest advances in related experimental research over the past five years. In conclusion, flavonoids can effectively ameliorate the pathological processes of multiple diseases by modulating autophagy pathways with favorable biosafety. Nevertheless, low bioavailability remains the core bottleneck restricting their clinical translation. Further optimization of pharmaceutical formulations is warranted to enhance their uptake efficacy in vivo, and rigorous clinical trials are needed to assess their prolonged effectiveness and potential drug interactions, so as to offer new feasible approaches and research directions for the prophylaxis and therapy of various diseases.

## 1. Introduction

Autophagy is a crucial biological mechanism through which cells break down and reuse intracellular proteins and subcellular structures to preserve cellular balance. This process exerts dual regulatory effects in a variety of diseases. Therefore, clarifying the specific functions of autophagy under pathological conditions serves as an essential precondition for the advancement of autophagy-directed interventions and therapies. Furthermore, autophagy is broadly implicated in the physiological modulation of cells and organisms. It not only enables cells to adapt to nutrient deficiency, sustain organelle homeostasis, and resist microbial infection, but also plays vital roles in innate immune responses, inflammatory reactions, and anti-aging processes. The term “autophagy” commonly refers to macroautophagy; apart from this, it also encompasses two other pathways, namely microautophagy and chaperone-assisted autophagy. During macroautophagy (hereinafter termed autophagy), evolutionarily preserved ATG complexes control the generation of double-membrane autophagosomes. After encapsulating cytoplasmic components, autophagosomes merge with lysosomes, thereby achieving the breakdown and repurposing of encapsulated substances [1,2,3].

Flavonoids constitute a group of naturally present polyphenolic substances widely found in plants, mostly in glycosylated forms. These compounds have long been shown to exhibit a variety of biological activities, such as antioxidant, anti-inflammatory, antimicrobial, antiviral, and antitumor effects, making them important candidate molecules for natural product research and mechanistic studies. Flavonoids are key active components in many Chinese medicinal herbs and are abundant in medicinal plants such as *Scutellaria baicalensis*, *Sophora japonica*, and *Pueraria lobata*. Existing studies have confirmed that certain flavonoids may play pharmacological roles in the prophylaxis and treatment of multiple disorders by modulating the autophagic process [4,5].

This is a narrative review aimed at summarizing the advances in research on the disease-modifying effects of flavonoids through the regulation of autophagy, based on a comprehensive overview and evaluation of existing investigations.

Literature search strategy: The literature search was conducted in PubMed and Web of Science using the keywords “flavonoids”, “autophagy”, “pharmacological effects”, and “disease treatment”. The search covered the past five years, with no language restrictions.

Inclusion criteria: (1) Original research articles or reviews on the pharmacological effects of flavonoids; (2) studies on flavonoid-regulated autophagy for disease treatment; (3) in vitro, animal, or clinical data; (4) published within the past five years.

Exclusion criteria: (1) Duplicate publications, conference abstracts, short communications, or incomplete data; (2) irrelevant to the review topic.

Literature organization and analysis: In vitro studies, animal experiments, clinical studies, and review articles were distinguished during literature organization. In the main text, findings are summarized by flavonoid subclasses (e.g., flavones, flavonols, dihydroflavonols). For each representative compound, in vitro and animal results are described separately. Clinical studies on flavonoid-regulated autophagy remain limited.

Due to the methodological limitations inherent in narrative reviews, the reference selection in this paper is primarily derived from the tracking of key publications. Consequently, certain selection bias may exist, making it difficult to comprehensively cover all relevant research outcomes in this area. Subsequent investigations may utilize the standardized protocols of systematic reviews to further standardize literature retrieval and screening strategies, thereby achieving more systematic and comprehensive evidence synthesis.

It is worth noting that, although numerous reviews have focused on autophagy regulation in disease therapy and the pharmacological effects of flavonoids, there has been no systematic review summarizing the scientific progress regarding the disease-modifying effects of flavonoid molecules through the regulation of autophagy. Therefore, the main added significance of this article is mainly manifested in three perspectives: firstly, focusing on the intrinsic molecular mechanisms of autophagy-mediated disease improvement; secondly, synthesizing current preclinical research findings to systematically elucidate the current status of different flavonoid subclasses (such as flavones, flavonols, and dihydroflavones) in intervening in diseases via autophagy regulation; and thirdly, addressing the common issue of low bioavailability of flavonoids and proposing targeted research and development strategies, as well as future perspectives.

Current research on the therapeutic effects of flavonoids in disease through autophagy regulation still has many shortcomings at the mechanistic level. For instance, the modulatory actions of different flavonoid subclasses on autophagy vary significantly across diseases and pathological stages. Most existing studies are limited to a single subclass and a single disease, and there is still a lack of systematic elucidation of their specific regulatory mechanisms and dose- and time-dependent patterns. Furthermore, research has mostly focused on the canonical autophagy pathway, while the multilevel and networked molecular regulatory mechanisms remain insufficiently revealed. Existing studies are predominantly preclinical, with inconsistent dosages and administration routes, leading to poor comparability and reproducibility of results. In addition, flavonoids themselves suffer from poor bioavailability and swift in vivo metabolism, resulting in a gap between in vitro and in vivo actions, and clinical application faces considerable obstacles.

This review systematically summarizes the diseases that are ameliorated by flavonoids via autophagy regulation, clarifies the activation or inhibition characteristics of flavonoids in regulating autophagy under different disease conditions, provides references for basic researchers and clinicians to quickly access applicable diseases of flavonoids, and analyzes existing research limitations as well as future research prospects.

## 2. Autophagy

**Figure 1:**
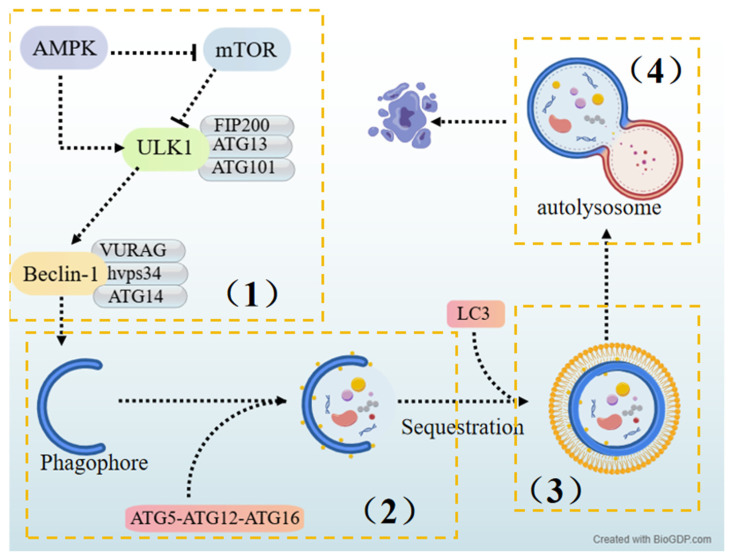
Autophagy regulation via the AMPK/mTOR signaling pathway. Created with BioGDP.com [11].

Autophagy is a preserved lysosome-dependent catabolic route within cells [6,7]. According to the distinct mechanisms of transporting intracellular substrates toward lysosomes, autophagy is primarily categorized into three forms: microautophagy, chaperone-mediated autophagy, and canonical autophagy. Macroautophagy is a strongly preserved degradation pathway where intracellular substrates are enclosed via bilayer autophagic vesicles and delivered into lysosomes for degradation. In contrast, microautophagy and chaperone-mediated autophagy do not require autophagosome formation; they primarily rely on the immediate catabolic role of lysosomes/vacuoles to achieve substrate elimination [8]. The cellular process of autophagosome biogenesis is considered to be essentially identical in non-selective and selective autophagy. Selective autophagy can be categorized into mitophagy, endoplasmic reticulum-phagy, lysophagy, nucleophagy, xenophagy, bacteriophagy, lipophagy, and glycophagy. As shown in Figure 1, inhibition of mTOR and stimulation of AMPK collectively facilitate autophagy [9]. mTOR acts as a suppressor of autophagy downstream of AMPK, and its activation inhibits autophagy. In nutrient-abundant environments, mTOR associates with and phosphorylates the ULK1 complex, thereby suppressing its kinase activity; under nutrient-deprived conditions, mTOR dissociates from the ULK1 complex, thereby activating autophagy [10].

Autophagy is regulated by a series of autophagy-related genes (ATGs). As shown in Figure 1, the progression of autophagy can be separated into four stages. (1) Commencement and phagophore formation: AMPK suppresses the assembly of the mTORC1 complex, thereby attenuating the suppressive action of mTORC1 on the generation of the ULK1 complex and facilitating the generation of autophagic vacuoles. The ULK1 complex assembly initiates phagophore nucleation by phosphorylating class III PI3K complex I. (2) Phagophore elongation: The ATG5-ATG12-ATG16 polymer complex fuses with the autophagic vesicle and inserts into the autophagosome via a cascade of events [12]. Full-length LC3 is processed by the cysteine proteinase ATG4, exposing the C-terminal glycine 120 [13]. Cysteine proteinase ATG4 processes LC3 to LC3-I, which is subsequently converted into membrane-associated LC3-PE with the help of two ubiquitin-like enzyme cascades (ATG3, ATG7). LC3 is catalyzed by ATG3, ATG7, and phosphatidylethanolamine to generate LC3-II. Subsequently, LC3-II enters the phagophore [14]. (3) Cargo encapsulation: LC3-PE is inserted into the double membrane of the autophagic vesicle, serving as a bridge during selective autophagy. It interacts with cargo adapters (such as p62, NBR1, and NDP52) to transport cargo to the autophagosome, thereby playing a role in autophagic selectivity [15]. LC3, a core autophagy protein, is the most widely used autophagy marker [16]. (4) Autophagosome-lysosome fusion: Lysosomes fuse with the external membrane of fully formed autophagosomes, discharging acidic hydrolases to degrade autophagic cargo for recycling intracellular nutrients and metabolic wastes. The autophagolysosome is the end product of autophagy [17].

## 3. Flavonoids

Flavonoids, a major class of plant secondary metabolites, exist as water-soluble pigments in plant cell vacuoles and are plentiful in fruits, vegetables, and herbs. They are indispensable in the nutraceutical and pharmaceutical fields. Modern pharmacology confirms that flavonoids possess anticancer, antioxidant, anti-inflammatory, and anti-atherosclerotic activities, showing great prospects for drug and functional food development. More than 10,000 flavonoid compounds have been separated and characterized to date [18,19,20]. Flavonoids possess a C6-C3-C6 carbon skeleton, with two six-carbon benzene rings (A and B) linked by a three-carbon heterocyclic C ring [21]. Flavonoids are categorized into seven subgroups according to alterations in their core skeletons; these categories comprise flavones, flavonols, flavanones, dihydroflavonols, flavanols, anthocyanins, and chalcones [22]. Most flavonoids can modulate autophagy, and the following sections will introduce representative flavonoids that regulate autophagy and their main characteristics (Table 1).

### 3.1. Flavones

Typical representatives of flavones include apigenin, luteolin, and baicalein, which are mainly found in celery, chamomile, and Ginkgo biloba leaves, and related studies have been extensive.

Apigenin is a natural flavonoid mainly obtained from genera of the Asteraceae family, including Tanacetum, Achillea, Artemisia, and Matricaria [23]. In plants, apigenin is present as nonanone and its C- and O-glycosides, glucuronides, O-methyl ethers, and acetyl derivatives [24]. Pharmacological studies have confirmed that apigenin possesses various biological activities, including inhibition of apoptosis [25], activation of autophagy [26], anti-inflammatory effects [27], regulation of microglia [28], and anti-atherosclerotic effects [29].

Luteolin is one of the most active flavonoid compounds among plant secondary metabolites [30]. It is extensively found in flowers and spices [31]. Structurally, luteolin is a 15-carbon flavone consisting of two benzene rings and one oxygen heterocycle, organized in the classic flavonoid structure mentioned earlier. Being a tetrahydroxyflavone, it has two hydroxy substituents on both of its aromatic rings [32]. The pharmacological effects of luteolin include alleviating liver injury [33], improving mitochondrial function [34], anticancer [35], antioxidant [36], anti-fibrotic [37], and anti-inflammatory [38] activities.

Baicalein, with the chemical formula C_21_H_18_O_11_, is an important flavonoid present in *Scutellaria baicalensis* [39,40]. This compound is widely used as an ingredient in herbal tea to promote health and has been studied for its various biological effects [41]. Its broad biological activities include anticancer [42], regulation of autophagy [43], improvement of insulin resistance [44], antiepileptic [45], regulation of gut microbiota [46], and anti-fibrotic [47] effects.

### 3.2. Flavonols

Flavonols represent a group of plant-specific metabolites that play key functions in plant growth and development. Flavonols differ from other flavonoids in their hydroxylation patterns on the benzene rings. Each flavonol has a unique hydroxylation pattern on its benzene rings. The most studied flavonols are quercetin, kaempferol, and rutin, which are widely found in fruits, tea, and cocoa [48,49].

Quercetin is an organic compound widely found in various fruits, nuts, and vegetables [50]. This compound possesses a broad range of medical properties and health benefits [51], mainly by acting on different protein targets, thereby exhibiting diverse biological functions. Compared with other flavonoids, quercetin is a potent antioxidant and is considered one of the most important flavonoids in the dietary and medical fields [52]. Furthermore, quercetin shows promise in the prophylaxis and treatment of multiple disorders, such as ameliorating diabetic nephropathy [53], inducing mitochondrial apoptosis [54], exerting anti-inflammatory [55] and anticancer effects [56].

Kaempferol ranks among the most extensively investigated flavonoids, having a small biochemical mass (286.2 g/mol) [57], and is present in various herbs and plant families. This compound is commonly present in traditional medicinal materials such as *Sophora japonica* and *Lycium chinense* [58]. As a polyphenolic molecule, kaempferol possesses the basic skeleton of phenylbenzopyran: Ring A is fused with heterocyclic ring C and linked to ring B. Its potent antioxidant activity is mainly ascribed to the phenolic hydroxyl groups on the aromatic rings [59]. Kaempferol possesses good biological activity [60] and exhibits diverse pharmacological properties, such as anti-inflammatory [61], antioxidant [62], lipid-reducing [63], anti-atherosclerotic [64], anti-apoptotic [65], ameliorative effects on acute myocardial infarction [66], and anti-fibrotic [67] effects.

Rutin is a citrus flavonoid glycoside [68] and represents a lipophilic moiety that dissolves in organic media like pyridine, methanol, and ethanol [69]. This agent exhibits various pharmacological properties, such as anti-inflammatory actions [70], anticancer [71], regulation of gut microbiota [72], and anti-oxidative stress [73] effects.

### 3.3. Flavanones

Flavanones possess a C15 skeleton with a C2–C3 single bond and C2 chirality. These compounds mostly exist in free or glycosidic forms in plants such as citrus, licorice, and hawthorn, with representative components including hesperetin and liquiritigenin [74,75].

Hesperetin is a naturally occurring phenolic substance. It is present in citrus species such as oranges and grapefruits and is considered safe with no obvious side effects [76,77]. Its broad biological activities include regulation of the gut microbiota [78], modulation of autophagy [79], and anti-oxidative stress [80].

Liquiritigenin is an important phytochemical found in various plants, with licorice being its best-known source [81]. As a potent antioxidant, liquiritigenin plays a critical role in scavenging accumulated free radical species and mitigating oxidative burden [82]. In addition, this compound exhibits various pharmacological activities, including modulation of autophagy [83], anticancer [84], and anti-fibrotic [85] effects.

### 3.4. Dihydroflavonols

Dihydroflavonols are a key subcategory of flavonoids and are widely present in diverse medicinal plants, vegetables, and fruits, such as conifers and grapes, mostly in free or glycosidic forms. Common representative compounds include silymarin and taxifolin (dihydroquercetin) [86].

Silymarin, a polyphenolic flavonoid extract from milk thistle seeds, consists mainly of 70–80% flavonolignans, 20–35% fatty acids, and other polyphenols [87]. This extract possesses various pharmacological activities, including anticancer [88,89], antioxidant [90], hepatoprotective [91], and anti-inflammatory [92] effects.

Taxifolin (dihydroquercetin) is a unique biologically active flavonoid widely distributed in species like olives and grapes. Its pharmacological activities originate from its hydroxylation pattern, structural type, substituents, and conjugates, as well as the degree of polymerization and metal chelation activity [93], including anti-fibrotic [94], regulation of the gut microbiota [95], antioxidant [96], and anti-inflammatory [97] effects.

### 3.5. Flavanols

Flavanols represent a flavonoid subclass, with common representatives including catechin, epicatechin, and proanthocyanidins.

Catechins are a group of naturally occurring polyphenolic substances. Compared with black tea, the catechin content in green tea is significantly higher [98,99]. Catechin content differs by tea type, with the chief constituents being catechin, epicatechin, epicatechin-3-gallate, and gallocatechin [100]. The pharmacological activities of catechins include improving lipid metabolism [101], anti-inflammatory [102], anti-fibrotic [103], and antihypertensive [104] effects.

Proanthocyanidins are widely present in the fruits and leaves of many plants, with their total content in grape seeds reaching as high as 99% [105]. Proanthocyanidins have few side effects and exhibit various bioactive properties, including improving lipid metabolism [106], antioxidant activity [107], inhibiting apoptosis [108], and anti-inflammatory [109] effects.

### 3.6. Anthocyanins

Anthocyanins, flavonoids with strong antioxidant activity, offer multiple health benefits; over 600 have been identified in plants [110], with the most common types including cyanidin, delphinidin, and others [111].

Research on anthocyanin derivatives has received widespread attention. Among them, cyanidin is an important bioactive component. Numerous studies have confirmed that anthocyanins (especially cyanidin) possess multiple therapeutic potentials [112], including anti-fibrotic [113], regulation of gut microbiota [114], antioxidant [115], reduction in pulmonary arterial hypertension [116], and anti-inflammatory [117] effects.

The structure of delphinidin is represented by a benzopyrylium cation skeleton, with hydroxyl substituents at both the R1 and R2 positions [118]. This compound exhibits pharmacological activities including anticancer [119], antioxidant [120], modulation of autophagy [121], and anti-inflammatory [122] effects.

### 3.7. Chalcones

Chalcones, a flavonoid subclass of phenolic compounds, are among the largest groups of bioactive natural products [123]. Widely distributed in plant bark, leaves, and roots [124], they possess anti-inflammatory [125] and anticancer [126] pharmacological activities.

In summary, based on the data in Table 1, the pharmacological activities of various flavonoids are generally dependent on dosage, animal models, and cell types. C57BL/6 and BALB/c mice, as well as SD and Wistar rats, are widely adopted for in vivo experiments, while genetically modified animals are used in some studies. The effective dose of an identical monomer varies markedly across diseases; for instance, kaempferol exerts anti-inflammatory effects at 50 mg/kg, yet a markedly higher dose is required in anti-apoptosis models. In vitro assays feature an extensive concentration range: hesperetin is pharmacologically active at concentrations as low as 0.005 μg/mL, whereas certain flavonoid constituents function at millimolar concentrations. Moreover, cells derived from different origins display distinct sensitivities when treated with the same flavonoid compound. Baicalein and luteolin exhibit consistent in vitro and in vivo pharmacological effects in cellular and animal assays, supporting further pharmacological investigation. In contrast, apigenin and other related monomers present divergent pharmacodynamic properties: low in vivo doses produce anti-inflammatory effects, whereas substantially higher concentrations are required to trigger autophagy in vitro. Such discrepancies may result from in vivo drug absorption, metabolism, and organ-targeted distribution, as well as the absence of physiological microenvironments, including body fluids and microcirculation in cultured cells. Future studies will establish reasonable in vitro-to-in vivo dose conversion criteria and optimize the selection of experimental models based on accumulated experimental data.

**Table 1:** Biological and pharmacological activities of flavonoids.

Category	HCA	Structure	Source	Biological Activity/Application	Experimental Model	Dose Range	Ref.
Flavones	Apigenin	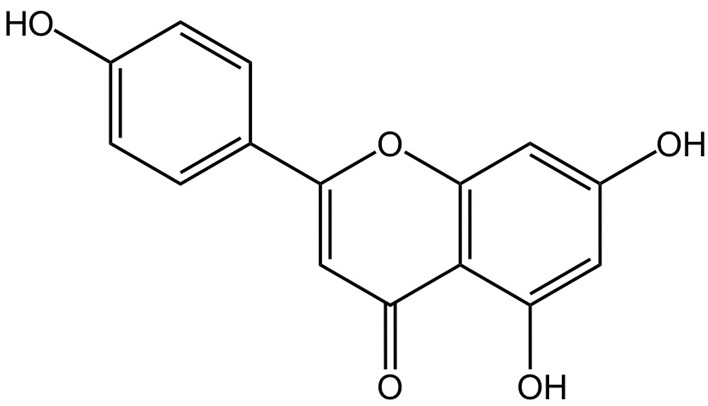	Dried parsley	Inhibition of apoptosis	B6SJL-Tg transgenic mice	40, 80 mg/kg	[25]
					NSC34 cells	1 μM	
				Activation of autophagy	Huh7, Huh7.5, HepG2, AML12 cells	0, 2.5, 5, 10, 20, 40, 80, 160 μM	[26]
				Anti-inflammation	C57/BL6 mice	50 mg/kg	[27]
					Caco-2 cells	10 μM	
				Regulation of microglia	CD-1 mice	25, 50, 75 mg/kg	[28]
				Anti-atherosclerosis	NLRP3−/−Ldlr−/− mice	50 mg/kg/day	[29]
	Luteolin	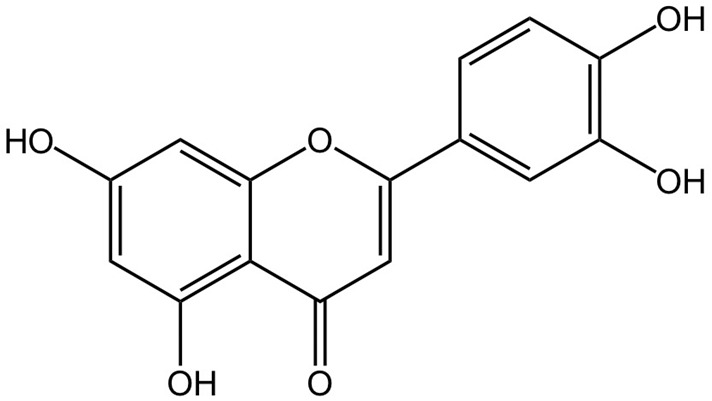	Honeysuckle flower	Alleviation of liver injury	C57BL/6 mice	40 mg/kg	[33]
					HepG2 cells	0, 2.5, 5, 10, 20, 30, 40, 80, and 100 μM	
				Improvement of mitochondrial function	Wistar rats	150 mg/kg/day	[34]
				Anticancer	BALB/c nude mice	20, 40 mg/kg	[35]
					ESCC cells	10, 20, 40, 80, and 120 μM	
				Antioxidant	SD rats	50, 100 mg/kg/day	[36]
				Anti-fibrotic	Wistar rats	10, 50 mg/kg	[37]
					HSC-T6 cells	10 μM	
				Anti-inflammatory	C57BL/6 mice	10, 20, and 50 mg/kg	[38]
					H9c2 cells	10, 20, 50 μM	
	Baicalein	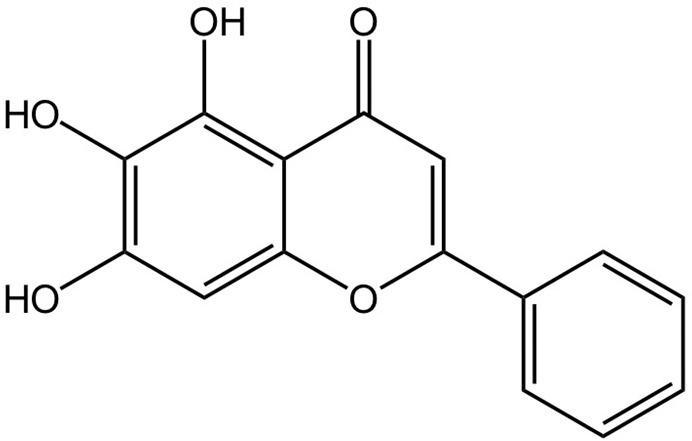	*Scutellaria baicalensis* (Huangqin)	Anticancer	C57BL/6 mice	50, 100, and 200 mg/kg	[42]
					H1299, A549 cells	0, 10, 20, 30, 50, 100, 200, and 500 μM	
				Regulation of autophagy	SD rats	100 mg/kg	[43]
				Improvement of insulin resistance	Glp1r KO mice	50, 100, and 200 mg/kg	[44]
					HepG2, C2C12 cells	40 μM	
				Antiepileptic	Sprague-Dawley (SD) rats	50, 100 mg/kg	[45]
					HT22 cells	0, 5, 10, 20 μM	
				Regulation of gut microbiota	C57BL/6J mice	10 mg/kg and 20 mg/kg	[46]
				Anti-fibrotic	C57BL/6J mice	25, 50, or 100 mg/kg	[47]
					CCC-ESF-1 cells	5–120 μM	
Flavonols	Quercetin	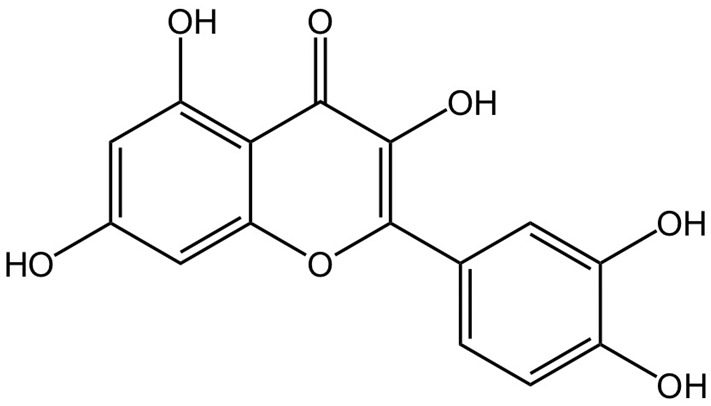	Onion	Amelioration of diabetic nephropathy	SD rats	100 mg	[53]
					HK-2 cells	0 μM, 5 μM, 10 μM, 15 μM, 25 μM, and 50 μM	
				Induction of mitochondrial apoptosis	SK-MEL-28, G-361 cells	0–400 μM	[54]
				Anti-inflammatory	A549 cells	0, 10, 20, and 40 μM	[55]
				Anticancer	ZR-75-1, MCF-7, T47D, MDA-MB-231, MCF10A, and MDA-kb2 cells	0, 5, 10, 20 μM	[56]
	Kaempferol	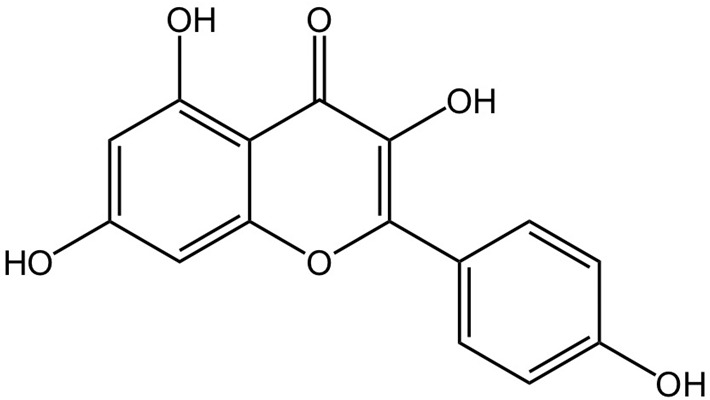	Broccoli	Anti-inflammatory	C57BL/6J mice	50 mg/kg/day	[61]
				Anti-oxidative stress	Sprague-Dawley rats	3, 10, or 20 mg/kg	[62]
				Reduction in lipid accumulation	HepG2 cells	5, 10, and 20 μM	[63]
				Anti-atherosclerotic	C57BL/6, APOE–/– mice	100 mg/kg	[64]
					HAECs cells	5, 10, or 20 μM	
				Anti-apoptotic	Albino rats	250 g/kg	[65]
				Amelioration of acute myocardial infarction	C57/BL6 mice	50 mg/kg/day	[66]
					H9c2 cells	0, 5, 10, and 20 μM	
				Anti-fibrotic	C57BL/6 mice	25, 50, and 100 mg/kg	[67]
					AML12 cells	5, 10, and 20 μM	
	Rutin	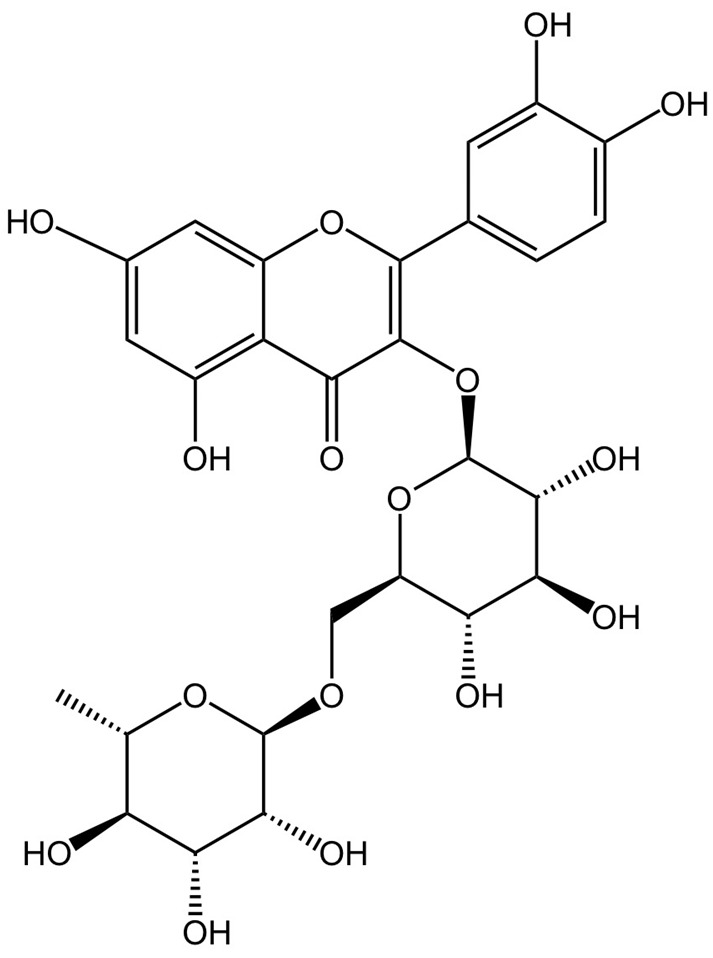	*Sophora japonica* (Huaimi)	Anti-inflammatory	HaCaT cells	0, 1, 5, 10, 15, 30, 50, and 100 μM	[70]
				Anticancer	PANC-1, SW1990, MIA PaCa-2 cells	5–40 μg/mL	[71]
				Regulation of gut microbiota	C57BL/6J mice	200 mg/kg	[72]
				Anti-oxidative stress	BALB/c mice	35 mg/kg	[73]
					RK-13, MDBK cells	10, 20, 40, 80, 160, and 200 μM	
Flavanones	Hesperetin	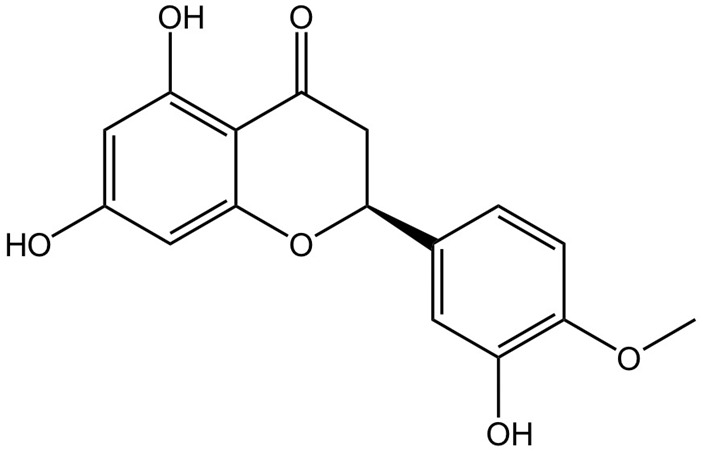	Orange	Regulation of gut microbiota	C57BL/6 mice	100 mg/kg/day	[78]
					RAW264.7 cells	0.005, 0.01, 0.05, 0.1, 0.2, 0.3, 0.5, 1, 2, and 5 μg/mL	
				Modulation of autophagy	U937, HL-60 cells	0, 12.5, 25, 50, and 100 μM	[79]
				Anti-oxidative stress	MH-S cells	10, 50 μM	[80]
	Liquiritigenin	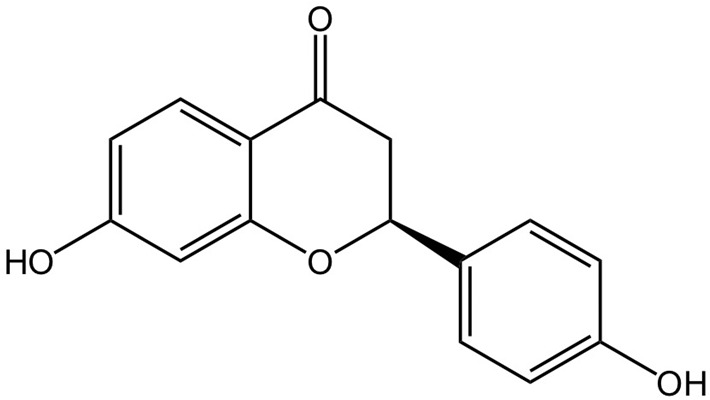	Glycyrrhiza uralensis (Ural licorice)	Modulation of autophagy	C57BL/6 mice	30 mg^−1^ kg^−1^ day^−1^	[83]
					MC3T3-E1 cells	0, 0.5, 1, 5, and 10 μM	
				Anticancer	MCF-7, BT20 cells	0, 0.05, 0.1, 0.2, 0.4, and 0.8 mmol/L	[84]
				Anti-fibrotic	C57BL/6 mice	25, 50, 100 mg/kg	[85]
					Pulmonary fibroblasts	0, 1, 3, 10, 30, and 100 μM	
Dihydroflavonols	Silybinin	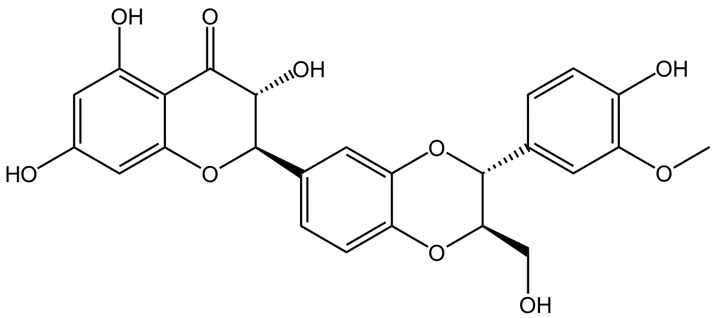	Silybum marianum (Milk thistle)	Anticancer	RPMI 8226, H929 cells	0–120 μM	[88]
					HepG2 and Hep3B cells	20 μmol/L, 40 μmol/L, and 80 μmol/L	[89]
				Anti-oxidative stress	Swiss Webster mice	50 mg/kg	[90]
				Hepatoprotective	Sprague-Dawley rats	50, 100, 200 mg/kg	[91]
				Anti-inflammatory	RAW264.7 cells	0.4, 0.8 μg/mL	[92]
	Taxifolin (Dihydroquercetin)	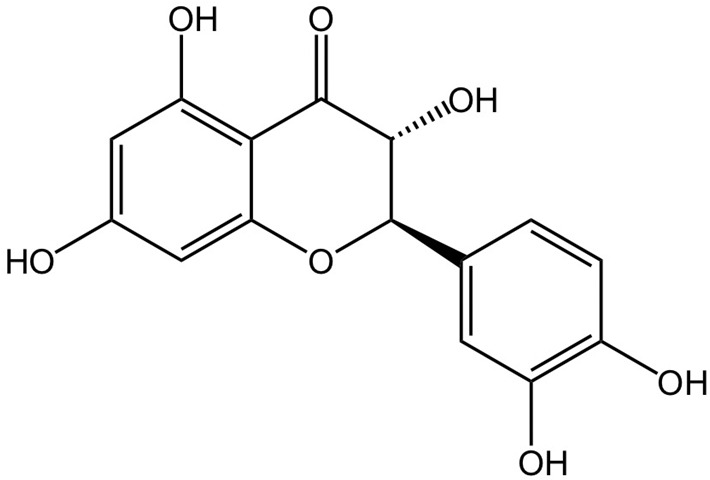	Siberian larch	Anti-fibrotic	C57BL/6 mice	10, 50 mg/kg	[94]
					HBE, MRC-5 cells	0, 5, 10, 20, 40, or 80 μM	
				Regulation of gut microbiota	C57BL/6J mice	50 mg/kg	[95]
				Antioxidant	SHR, Wistar rats	25, 50 mg/kg/d	[96]
					PC-12 cells	0.1, 0.5, 1 nM	
				Anti-inflammatory	BV-2 cells	200, 400 μM	[97]
Flavanols	Catechin	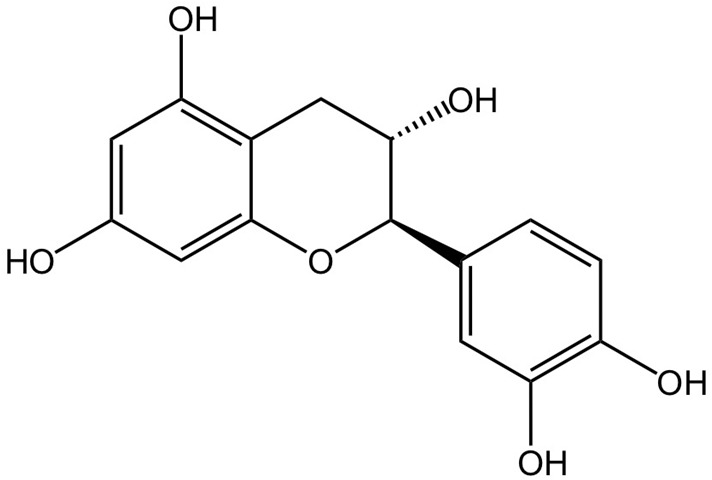	Tea leaves	Improvement of lipid metabolism	C57BL/6 mice	1328, 2645, 5289 mg/kg	[101]
				Anti-inflammatory	SD rats	0.5, 1, 1.5, and 2 mg/kg	[102]
				Anti-fibrotic	C57BL/6 mice	50 μg/kg	[103]
					PSCs cells	250 μM	
				Antihypertensive	SHR rats	10, 50 mg/kg	[104]
	Proanthocyanidins	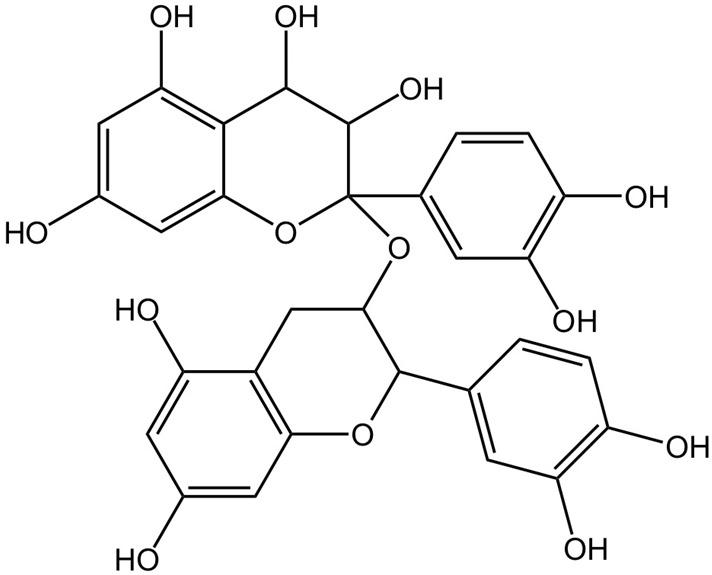	Grape seeds	Improvement of lipid metabolism	C57BL/6J mice	1, 2%	[106]
				Anti-oxidative stress	Kunming mice	200 mg/kg/day, 20 mg/kg	[107]
				Inhibition of apoptosis	SD rats	10, 20, 40 μM/mL	[108]
					MG63 cells	10 mg/kg	
				Anti-inflammatory	SD rats	200 mg/kg	[109]
Anthocyanins	Cyanidin	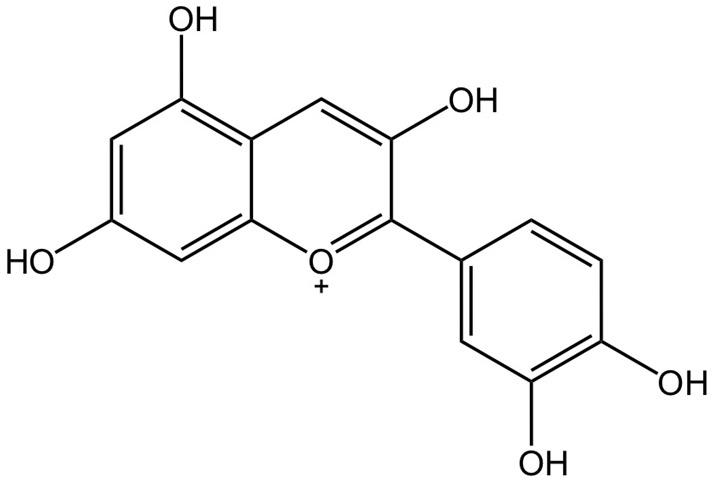	Blueberry	Anti-fibrotic	C57BL/6 mice	0, 50, 100, 150, 200 μmol/L	[113]
					HK-2 cells	100 mg/kg/day	
				Regulation of gut microbiota	C57BL/6J mice	10, 50 mg/kg	[114]
				Antioxidant	BALB/c male mice	60 mg/kg	[115]
				Reduction in pulmonary arterial hypertension	Sprague-Dawley rats	10 or 20 μM	[116]
					HPASMCs cells	40 mg/kg	
				Anti-inflammatory	Male BALB/c mice	100 mg/kg	[117]
	Delphinidin	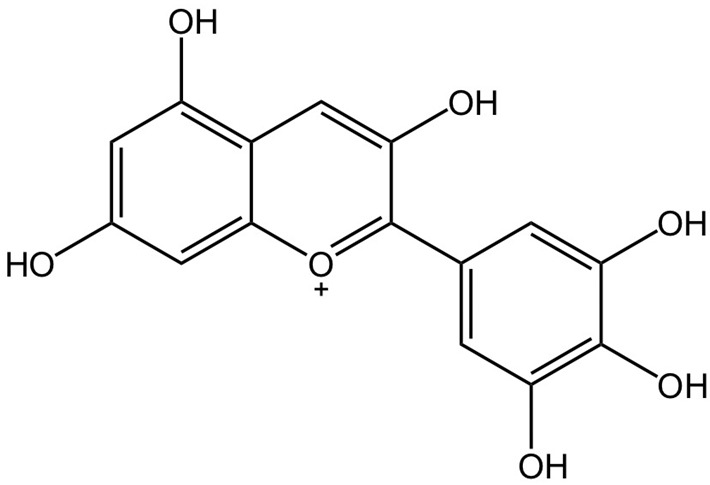	Blueberry	Anticancer	Sprague-Dawley rats	40 μmol/L	[119]
					MDA-MB-231, MCF-7, and MDA-MB-453 cells	2.6 mg/kg	
				Antioxidant	C57BL/6J mice	0–50 μM	[120]
				Modulation of autophagy	A549 cell line	10, 20 mg/kg/d	[121]
				Anti-inflammatory	New Zealand white rabbits	1.5 μM	[122]
Chalcones	——	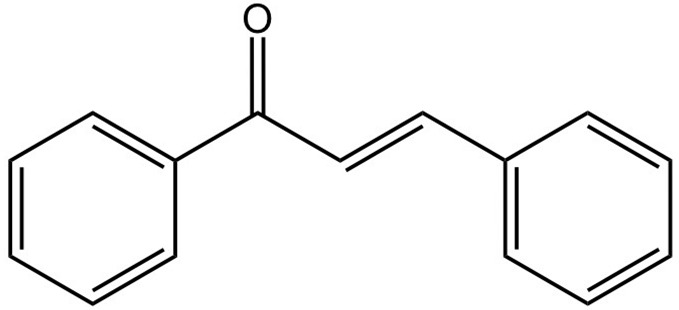	Asitaba (Angelica keiskei)	Anti-inflammatory	THP-1 cell line	20 μg/mL	[125]
				Anticancer	Luc-4T1, MDA-MB-231 cells	40, 80 mg/kg	[126]

## 4. Flavonoids Improve Diseases by Regulating Autophagy

**Figure 2:**
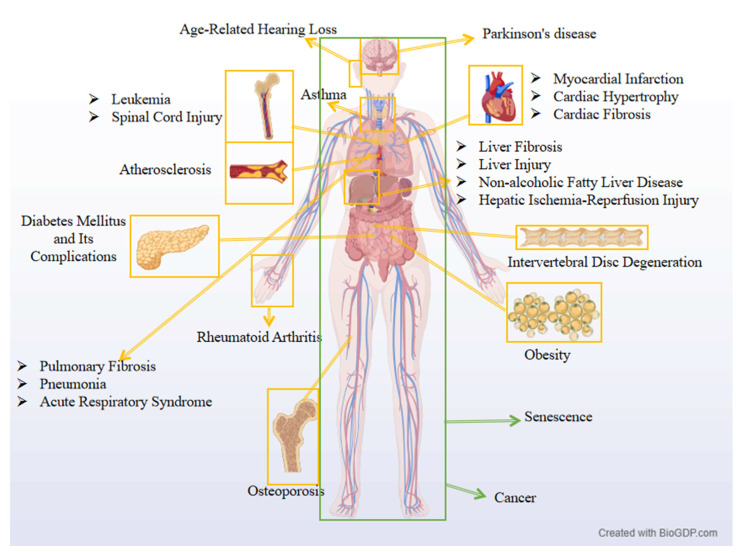
Flavonoids improve various diseases by modulating autophagy. Created with BioGDP.com [11].

Flavonoids represent a category of small polyphenolic molecules widely found in medicinal plants and natural food ingredients, offering advantages such as broad sources, high safety, and multi-target regulation [127,128]. As shown in Figure 2 and Table 2, recent studies have confirmed that flavonoids can exert multiple protective actions—comprising anti-inflammatory, antioxidative, anti-apoptotic, anti-fibrotic, and anti-cancer effects—across multiple systemic disorders such as atherosclerosis, intervertebral disk degeneration, liver fibrosis, myocardial ischemia–reperfusion injury, cancer, osteoporosis, diabetes, Parkinson’s disease, and non-alcoholic hepatic steatosis by regulating autophagy pathways. The core mechanism lies in modulating autophagic activity, restoring autophagy-related flux and the homeostasis of related signaling pathways, alleviating tissue damage, inhibiting abnormal proliferation, and mitigating inflammation and fibrosis, thereby achieving disease prevention and therapeutic effects. In-depth clarification of the targets and molecular pathways by which flavonoids regulate autophagy could offer a novel theoretical basis and experimental evidence for clinical disease management and the development of natural medicines.

### 4.1. Atherosclerosis

Atherosclerosis is a disease marked by the deposition and calcification of fats and fibrillar components in major arteries [129] and is a major cause of morbidity and mortality worldwide. Zixuan Hu et al. [129] demonstrated that apigenin effectively modulates serum and hepatic lipid levels and promotes autophagosome formation, thereby inhibiting the initiation of atherosclerosis.

### 4.2. Intervertebral Disk Degeneration

Intervertebral disk degeneration (IVDD) is an age-related global musculoskeletal disorder. Over 40% of low back pain cases stem from IVDD, which is induced by multiple pathological factors, including abnormal mechanical stress, aging, genetic susceptibility, and obesity. Chenglong Xie et al. [130] showed that apigenin protects nucleus pulposus (NP) cells against TBHP-triggered apoptosis, aging, and extracellular matrix (ECM) degradation in vitro by restoring autophagic flux, and also alleviates IVDD advancement in rats in vivo. Shuwen Zhang et al. [131] demonstrated that quercetin alleviates intervertebral disk degeneration by suppressing autophagy activation mediated by the p38 MAPK/mTOR signaling cascade. Md Entaz Bahar et al. [132] showed that delphinidin alleviates excessive reactive oxygen species (ROS)-overload-induced damage to human nucleus pulposus cells under oxidative conditions by activating the autophagy pathway, effectively inhibiting cell senescence and apoptosis and mitigating ECM degradation. The underlying mechanism involves the regulation of the ROS–AMPK–mTOR signaling axis, thereby ameliorating intervertebral disk degeneration.

### 4.3. Liver Diseases

Liver fibrosis represents a long-term pathological condition triggered by multiple factors and serves as a required phase for numerous liver disorders to progress to cirrhosis or even hepatocellular carcinoma. Jie Ji et al. [133] demonstrated that apigenin exerts hepatoprotective effects through mechanisms involving the inhibition of the TGF-β1/Smad3 and p38/PPARα signaling pathways, as well as by reducing autophagy and liver fibrosis formation. Muqing Zhang et al. [134] showed that liquiritigenin protects the liver against arsenic trioxide-induced injury through its antioxidant and anti-inflammatory properties, and upregulates autophagy through the PI3K/AKT/mTOR signaling cascade.

Non-alcoholic fatty liver disease (NAFLD) is typified by hepatic fat accumulation in the absence of additional clear causes of hepatic lipid storage, such as significant alcohol consumption. Ioannis Katsaros et al. [135] demonstrated that quercetin improves NAFLD by regulating the protein levels of Beclin-1, p62, and LC3. Fatemeh Mokhtari-Andani et al. [136] showed that silymarin supplementation attenuates abnormal mitophagic signaling within liver cells of dexamethasone-evoked NAFLD model rats, possibly offering hepatoprotection and preventing further injury. Zhilu Yao et al. [137] demonstrated that proanthocyanidins reduce hepatic ischemia–reperfusion injury via stimulating the PPARα/PGC1α signaling cascade and inhibiting autophagy and apoptosis. Qiao He et al. [138] demonstrated that cyanidin attenuates chronic-binge alcohol-evoked liver injury, in which activation of the AMPK/mitophagy axis serves a critical function in such protective actions.

### 4.4. Cardiac Diseases

Reperfusion therapy for acute myocardial infarction restores blood flow to ischemic myocardium and triggers myocardial ischemia–reperfusion injury (MIRI), resulting in adverse cardiac remodeling, reperfusion arrhythmias, and myocardial stunning. Chenchen Tian et al. [139] demonstrated that apigenin improves myocardial infarction by modulating autophagic and apoptotic processes through the miR-448/SIRT1 axis. Xiyan Dai et al. [140] showed that luteolin modulates biochemical and metabolic indicators, promotes autophagy, prevents cardiac hypertrophy and fibrosis, and thereby ameliorates myocardial injury. Bing-Yan Liu et al. [141] demonstrated that baicalein improves cardiac hypertrophy by activating FUNDC1 to enhance impaired autophagy. Jiqiang Hu et al. [142] showed that quercetin promotes autophagy by regulating the miR-223-3p/FOXO3 axis, thereby preventing isoproterenol (ISO)-induced myocardial fibrosis.

### 4.5. Cancer

Cancer is a broad term for cancerous diseases marked by uncontrolled proliferation, uncontrolled growth, and the ability to invade and metastasize, encompassing many common malignancies including lung, gastric, liver, and colorectal carcinomas [143]. Ling Wu et al. [144] demonstrated that luteolin promotes apoptosis and autophagy in TNBC cells through the SGK1-FOXO3a-BNIP3 axis, thereby inhibiting tumor growth. Bingjie Hao et al. [145] showed that baicalein promotes the interaction between CD274 and LC3, enhances autophagic degradation of CD274, boosts T-cell-mediated antitumor immunity, and inhibits lung tumorigenesis. Jie Zhang et al. [146] showed that quercetin inhibits non-small cell lung cancer by regulating mitochondrial autophagy, regulated by the SHP2/ROS/AMPK/XBP-1/DJ-1 signaling cascade. Baohong Yuan et al. [147] showed that silymarin may enhance the antitumor efficacy of doxorubicin by modulating autophagy, angiogenesis, and apoptosis. Yuting Chen et al. [148] demonstrated that CA13, a novel adamantyl-substituted chalcone derivative, exerts potent cytotoxic effects in lung cancer cells by inducing JNK-mediated apoptosis and protective autophagy, thereby inhibiting tumor growth and progression. Gina Mendez-Callejas et al. [149] showed that Chromolaena exerts dual suppressive actions on breast cancer cells in a cell-type-dependent manner: primarily activating mTOR-mediated autophagy in MCF-7 cells, while inducing mitochondrial apoptosis in MDA-MB-231 cells, along with G0/G1 phase blockade, demonstrating strong anti-breast cancer potential. Gina Mendez-Callejas et al. [150] demonstrated that 2′,3,4-trihydroxy-4′,6′-dimethoxychalcone exerts potent antiproliferative effects on cancer cells, particularly TNBC. It induces autophagy by regulating mTOR protein conformation and triggers caspase-3/7-dependent apoptosis, thereby presenting promising anticancer potential. M. Rossi et al. [151] showed that Licochalcone A displays anti-proliferative effects via triggering programmed cell death and autophagic processes, while also reducing cell invasion, thereby exerting antitumor effects.

### 4.6. Osteoporosis

Osteoporosis (OP) is the most prevalent degenerative bone disease, impacting millions of individuals globally. The diagnosis of OP is often challenging because it is largely asymptomatic and usually becomes definite only after a fracture occurs. Liang Tang et al. [152] demonstrated that in rats, enhancing the autophagic process through the miR-125b-5p/SIRT3/AMPK/mTOR pathway and upregulating RUNX2, OSX, OPN, and OCN levels promote osteogenesis and ameliorate OP. Yue Xiong et al. [153] showed that quercetin improves OP by inhibiting NLRP3-mediated production of inflammatory factors and suppressing osteoclast autophagy.

### 4.7. Diabetes and Its Complications

Diabetes mellitus is a metabolic disease primarily marked by elevated apoptosis and impaired function of pancreatic β-cells, resulting from impaired islet function. Ming Han et al. [154] reported that luteolin ameliorates pancreatic β-cell dysfunction by promoting autophagy and exerting antioxidant effects. Hongqin Sheng et al. [155] demonstrated that kaempferol reduces the urinary albumin-to-creatinine ratio (UACR), ameliorates glucosphingolipid metabolism disorders and podocyte injury in db/db mice, and alleviates diabetic nephropathy by modulating the AMPK/mTOR pathway. Reem Alshaman et al. [156] showed that hesperetin effectively alleviates the pathological changes in diabetic retinopathy by suppressing the inflammatory burden and inducing autophagy. Chao Tian et al. [157] demonstrated that epigallocatechin restores keratinocyte autophagy through the AMPK/ULK1 pathway, promotes keratinocyte and fibroblast activation, and thereby accelerates diabetic wound healing. Qiang Jia et al. [158] reported that epigallocatechin reduces cardiac fibrosis in type 2 diabetic rats; the process may entail stimulation of AMPK/mTOR-mediated autophagy, followed by inhibition of the TGF-β/MMPs cascade, thus decreasing excess collagen accumulation in the diabetic myocardium.

### 4.8. Parkinson’s Disease

Parkinson’s disease (PD) is a common neurodegenerative disorder marked by Lewy body formation and dopaminergic neuron loss in the substantia nigra. Min Chen et al. [43] demonstrated that baicalein stimulates mitophagy via the miR-30b-5p and SIRT1/AMPK/mTOR cascades, thereby exerting a protective effect in PD rats. Xiaojuan Han et al. [159] showed that kaempferol prevents dopaminergic neuron degeneration in PD by promoting lipophagy and inhibiting lipid peroxidation-mediated mitochondrial damage.

### 4.9. Asthma

Asthma is a chronic airway inflammatory disease characterized by bronchial hyperresponsiveness and reversible airflow obstruction. Liye Lang et al. [160] demonstrated that quercetin alleviates airway inflammation and lung injury in OVA-induced high-Th2 allergic asthma mice and restores excessive autophagy.

### 4.10. Age-Related Hearing Loss

Age-related hearing loss (ARHL) is a common age-related sensory disorder characterized by progressive sensorineural hearing loss in the elderly. Menglong Feng et al. [161] showed that quercetin potently alleviates ARHL in mice. Its protective effects are achieved by enhancing mitophagy and simultaneously inhibiting the NLRP3 inflammatory complex, thereby lowering oxidative burden and inflammatory reactions.

### 4.11. Leukemia

Leukemia is a global public health concern, and chemotherapy serves as the primary treatment for adult acute myeloid leukemia. Ching-Yeh Lin et al. [79] reported that hesperetin induces mild apoptosis, cell-cycle arrest, and autophagy to trigger cell death in human leukemia U937 cells. Thus, hesperetin may serve as a potential adjuvant for anti-leukemia therapy.

### 4.12. Pulmonary Diseases

Pulmonary fibrosis is an age-related disease with progressive lung function decline, high mortality, and limited therapeutic options. Qi Lin et al. [162] demonstrated that hesperetin exerts protective effects against lung fibrosis through Nrf2 pathway activation and alleviation of impaired autophagy in a CISD2-dependent manner. Aydin Genc et al. [163] showed that silymarin exerts remarkable protective actions against methotrexate-induced lung injury and may serve as an effective adjunctive therapy to mitigate chemotherapy-related pulmonary toxicity. Meili Shen et al. [164] demonstrated that epigallocatechin inhibits inflammation, oxidative stress, and apoptosis, and promotes autophagy, thereby ameliorating lung inflammation in a rodent model of experimental pneumonitis.

Severe acute respiratory syndrome (SARS) is a fatal SARS-CoV-induced disease with high morbidity and mortality, first reported in southern China and Hong Kong. Chih-Ching Yang et al. [165] showed that catechin directly inhibits SARS-CoV replication, enhances adaptive immunity, and alleviates ALI and cytokine storm via PI3K/AKT/mTOR-mediated autophagy, thus potentially preventing/treating SARS-CoV infection.

### 4.13. Obesity

Obesity features white adipose tissue expansion and morphological changes in adipocytes, mainly caused by excessive triglyceride accumulation. Hong Qin et al. [166] demonstrated that liquiritigenin inhibits fat build-up in 3T3-L1 white fat cells via the mTOR signaling pathway, suggesting that liquiritigenin is a promising naturally occurring bioactive ingredient in nutritional supplements used for obesity prevention.

### 4.14. Rheumatoid Arthritis

Rheumatoid arthritis (RA) is a long-term systemic inflammatory disorder marked by the destruction of bone and cartilage, primarily resulting from persistent synovial inflammation. Ye-Rin Heo et al. [167] demonstrated that proanthocyanidins inhibit cell viability and reduce ROS levels by triggering apoptosis and autophagy in RA-FLS, thereby playing an important role in RA treatment.

### 4.15. Spinal Cord Injury

Spinal cord injury (SCI) is a disabling disease causing permanent paralysis and multiple neurological dysfunctions, severely impairing patients’ quality of life and survival. Haojie Zhang et al. [168] showed that 3,4-dimethoxychalcone promotes TFEB nuclear translocation via the AMPK-TRPML1-calcineurin pathway, enhancing post-SCI autophagy and functional recovery.

### 4.16. Cellular Senescence

Cellular senescence is a permanent cell-cycle arrest involved in tissue remodeling, wound healing, immune defense, and tumor suppression, but may also disrupt homeostasis, impair regeneration, induce inflammation, and promote carcinogenesis. Based on the activation mechanism, it is classified into replicative, physiological, and stress-induced premature senescence. Nagarajan Maharajan et al. [169] demonstrated that Licochalcone D alleviates oxidative challenge-elicited early senescence and improves aging-related phenotypes by activating the AMPK pathway, modulating impaired autophagy, and reducing RAGE expression in hippocampal tissue.

**Table 2:** Pharmacological Effects and Mechanisms of Flavonoids in Ameliorating Diseases via Autophagy Regulation.

HCA	Disease	Promote/ Inhibit	Selective Autophagy/ Autophagy	Mechanisms of Action	Experimental Model	Dose Range	Ref.
Apigenin	Atherosclerosis	Activation	Lipophagy	UVRAG, Beclin1, PI3KC3, ATGs, ATG5, ATG3, LC3-II/I ↑	ApoE−/− mice	6.25, 12.5 mg/kg/d	[130]
	Intervertebral disk degeneration	Activation	Autophagy	Lamp2, p62 ↓, LC3-II/I, TFEB ↑	SD rats	10 mg/kg	[131]
					NP cells	0–50 μM	
	Liver fibrosis	Inhibition	Lipophagy	Beclin-1, LC3-II/I ↓, p62 ↑	C57 mice	20, 40 mg/kg	[134]
					LX2 cell line	10, 20, 30, 40, 50, 60, 70, or 80 μM	
	Myocardial ischemia–reperfusion	Activation	Mitophagy	SIRT1, LC3-II/I ↑, p62 ↓	H9c2 cells	10 μM	[139]
Luteolin	Triple-negative breast cancer	Activation	Autophagy	Beclin-1, LC3-II/I ↑, P62 ↓	BALB/c mice	20, 40 mg/kg	[144]
					TNBC cell line	0, 12.5, 25, 50, 100, 200 μM	
	Osteoporosis	Activation	Autophagy	Beclin-1, ATG5, LC3-II/I ↑, P62 ↓	SD rats	50, 100 mg/kg	[152]
					BMSC cells	1, 10, and 100 μM	
	Diabetes mellitus	Activation	Autophagy	DRAK2, P62 ↓, ULK1, LC3-II/I ↑	ICR mice	20 mg/kg	[154]
					INS-1E cells	20 μM	
	Myocardial injury	Activation	Autophagy	Beclin-1, LC3-II/I ↑, P62 ↓	Wistar rats	25, 50, 100 mg/kg	[140]
Baicalein	Cardiac hypertrophy	Activation	Mitophagy	LC3-II/I, FUNDC1 ↑	C57BL/6	25 mg/kg	[141]
					NRCMs cells	5–30 μM	
	Enhancement of antitumor immunity	Activation	Autophagy	CD274, LC3B ↑	C57 mice	33 mg/kg	[145]
					A549, H1299, 293T cells	25, 50, 100 μM	
	Parkinson’s disease	Activation	Mitophagy	p-AMPK/AMPK, LC3-II/I ↑, p-mTOR/mTOR, P62 ↓	SD rats	100 mg/kg	[43]
Quercetin	Myocardial fibrosis	Activation	Mitophagy	FOXO3, ATG7, LC3-II/I ↑, p62 ↓	Wistar rats	25 mg/kg	[142]
					293T, RCF cells	50 μM	
	Non-small cell lung cancer	Activation	Mitophagy	P-AMPK/AMPK, Beclin-1, PINK1, Parkin, NOX4, XBP-1, DJ-1 ↑, SHP2 ↓	SPF male nude mice	50 mg/kg	[146]
					AC16 cells	80 μM	
	Intervertebral disk degeneration	Inhibition	Autophagy	Beclin-1, LC3II/I, p38 ↑, p62, p-mTOR/mTOR ↓	SD rats	100 mg/kg	[132]
					NFC cells	15, 25 μM	
	Osteoporosis	Activation	Autophagy	Beclin-1, LC3II/I ↓, p62 ↑	SD rats	50 mg/kg	[153]
	Non-alcoholic fatty liver disease	Inhibition	Autophagy	Beclin1, LC3II/I ↑, p62 ↓	C57BL/6J mice	50 mg/kg	[136]
	Asthma	Activation	Autophagy	LC3-II/LC3-I, Beclin-1 ↓, p-PI3K/p-Akt, p-mTOR/mTOR ↑	BALB/c mice	10, 20 mg/kg	[160]
	Age-related hearing loss	Activation	Mitophagy	PINK1, PARKIN, BNIP3, LC3II/I ↑	C57BL/6J mice	50 mg/kg	[161]
Kaempferol	Parkinson’s disease	Promote	Mitophagy	LC3II/I ↑, p62 ↓	C57BL/6J mice	50 mg/kg	[159]
	Diabetic nephropathy	Promote	Autophagy	LC3II/I, Beclin-1, Atg5, Atg7 ↑, p62 ↓	C57BLKS/J db/db, db/m mice	50, 100 mg/kg/day	[155]
Hesperetin	Leukemia	Activation	Autophagy	LC3-II/I, Atg5, Beclin-1 ↑, p62 ↓	U937, HL-60 cells	0, 12.5, 25, 50, and 100 μM	[79]
	Pulmonary fibrosis	Activation	Autophagy	BECN1 ↑, BCL2 ↓	C57BL/6 mice	50 mg/kg/day	[163]
					A549 cells	10 μM	
	Diabetic retinopathy	Inhibition	Autophagy	LC3-II/I, Beclin-1 ↑, p62 ↓	Wistar rats	50, 100 mg/kg	[156]
Liquiritigenin	Liver injury	Promote	Mitophagy	p-PI3K/PI3K, p-AKT/AKT, p-mTOR/mTOR ↑	Kunming mice	20, 40 mg/kg	[135]
	Obesity	Promote	Lipophagy	LC3-II/I, ATG7 ↑, p62, p-mTOR/mTOR ↓	3T3-L1 cells	10, 20, 25 nM	[167]
Silymarin	Hepatocellular carcinoma	Inhibition	Autophagy	Beclin-1, LC3-I/LC3-II ↑	H22 cells	0, 40, and 80 μg/mL	[147]
	Non-alcoholic fatty liver disease	Inhibition	Mitophagy	Parkin, Bcl-2, LC3-II/I, PINK1, p62 ↓	Wistar rats	300 mg/kg	[137]
	Lung injury	Promote	Autophagy	LC3-II/I, Beclin-1 ↓	Wistar rats	50 mg/kg	[164]
Catechin	Diabetic skin ulcer	Promote	Autophagy	LC3-II/I, Beclin1, ATG5, p-AMPK/AMPK, p-ULK1/ULK1 ↑, p62 ↓	SD rats	1 mg/mL	[157]
					HaCaT, HFF-1 cells	0, 6.25, 12.5, 25, 50, 100 μM	
	Diabetic cardiomyopathy	Promote	Autophagy	LC3, Beclin1, p-AMPK/AMPK ↑, p-mTOR/mTOR ↓	SD rats	40, 80 mg/kg	[158]
	Pneumonia	Promote	Autophagy	LC3, BECN1 ↑, mTOR ↓	Wistar rats	15 mg/kg	[165]
	Severe acute respiratory syndrome (SARS)	Inhibition	Autophagy	Beclin-1, Atg5, Atg12, LC3-II ↑	BALB/c mice	25, 50, 125 mg/kg	[160]
					Vero E6 cells	6.25 mg–195 μg	
Proanthocyanidins	Hepatic ischemia–reperfusion injury	Promote	Autophagy	LC3, Beclin-1 ↓, Bcl-2 ↑	BALB/c mice	50 mg/kg, 50 mg/kg, 100 mg/kg	[161]
	Rheumatoid arthritis	Promote	Mitophagy	LC3-II/I ↑, p62 ↓	RA-FLS cells	0 to 400 μg/mL	[162]
	Alcohol-related liver disease	Promote	Autophagy	p62, LC3-II ↓, Parkin, p-AMPK/AMPK ↑	C57BL/6J mice	50 μg/kg	[163]
					AML12 cells	1 μg/mL	
Delphinidin	Intervertebral disk degeneration	Promote	Autophagy	LC3-II/LC3-I, Beclin-1, p-AMPK/AMPK, SIRT1 ↑, p62, p-mTOR/mTOR ↓	hNPCs cells	0 to 200 μM	[164]
Chalcone	Spinal cord injury	Promote	Autophagy	VPS34, Beclin1, LC3-II/LC3-I ↑, CTSD ↓	C57BL/6J mice	0, 100, 150, 200, 250 mg/kg/day	[165]
	Lung cancer	Promote	Autophagy	LC3-II ↑	BALB/c-nu/nu mice	20, 40 mg/kg	[148]
					A549, H460, H292, MRC-5 cells	0, 2.5, 5 μM	
	Breast cancer	Promote	Autophagy	LC3-II, Beclin-1 ↑, p62, p-mTOR ↓	MDA-MB-231, MCF-7 cells	5–80 μg/mL	[149]
	Cancer	Activation	Autophagy	LC3-II, p53 ↑, p62, mTOR ↓	TNBC MDA-MB-231, SiHa, MCF-7, PC-3 cells	1–10 μM	[150]
	Osteosarcoma	Activation	Lipophagy	LC3-II ↑, p62 ↓	MG63, 143BOS cells	80, 40, 20, 10, 5, 2.5, 1.25, 0.6125 μM	[151]
	Aging/Cellular senescence	Inhibition	Autophagy	LC3-II, Beclin-1, p-AMPK/AMPK ↑, p62 ↓	C57BL/6 mice	0.5 mg/kg/day	[170]
					hBM-MSCs cells	0–8 μg/mL	

## 5. Bioavailability and Safety of Flavonoids

Bioavailability is a core indicator for evaluating the development value of oral formulations and directly determines the clinical application potential of flavonoids. Due to their inherent chemical structural limitations, most flavonoids generally suffer from poor water solubility, insufficient membrane permeability, and rapid metabolism, leading to low oral bioavailability. This not only restricts their clinical translation but also directly impacts their safety and therapeutic efficacy. Representative flavonoids such as apigenin, luteolin, and kaempferol exhibit varying degrees of absorption limitations: apigenin has extremely poor water solubility; following oral dosing, it is primarily taken up in the intestinal tract and eliminated slowly from the body, requiring formulation optimization to improve its systemic exposure [170]; although luteolin has significant pharmacological activity, its oral absorption is poor due to first-pass metabolism [171]; the bioavailability of kaempferol is affected by the type of glycosyl group and intestinal hydrolysis processes; after hepatic metabolism, it is excreted in the urine without obvious safety concerns [57]. The metabolic characteristics of baicalein and quercetin differ: baicalein has a rapid metabolic rate, with very low levels of the parent compound in the circulation, mainly existing as conjugated metabolites [172]; quercetin is highly hydrophobic, tends to precipitate in the gastrointestinal environment, and has low bioaccessibility [173]; the oral bioavailability of delphinidin glycosides and catechins is also relatively low, and formulation optimization is likewise needed to improve their absorption [99,117]. The overall oral bioavailability of silymarin is low: in rats, the oral utilization rate is only about 0.95%; in healthy humans, only 10–17% is present in free unconjugated form after oral administration, and most is rapidly converted by phase II metabolic enzymes into conjugated products such as glucuronides and sulfates, with rapid metabolism, a short half-life, and plasma concentrations typically at the nanomolar level [174]. Liquiritigenin’s low oral bioavailability stems from poor water solubility and low membrane permeability, causing limited absorption and rapid metabolism [81].

To address the above issues, advanced formulation technologies such as liposomes and nanocarriers, combined with solid dispersion and local administration, are currently employed to reduce gastrointestinal degradation and first-pass effects, thereby improving the absorption efficiency and stability of flavonoids. Overall, enhancing the bioavailability of flavonoids still requires overcoming formulation translation bottlenecks. At the same time, leveraging their favorable safety profiles, further efforts should be made to promote their clinical translation from basic research, thereby fully realizing their pharmacological potential.

## 6. Summary and Perspectives

Most previous reviews focus on individual flavonoids or a single type of disease. In contrast, this review is distinguished by systematically summarizing the functions of various flavonoid subclasses in multiple diseases via regulating autophagy pathways. For the first time, we comprehensively elaborate on the protective effects of flavonoids against a broad spectrum of disorders, including atherosclerosis, degenerative diseases, metabolic diseases, tumors, and neurological diseases. Furthermore, we discuss key bottlenecks in clinical translation, such as dose extrapolation and formulation optimization, and propose targeted research and development strategies. These topics have rarely been addressed in earlier reviews.

Flavonoids are a class of structurally diverse, widely sourced natural polyphenols that exert multi-target protective and therapeutic effects in various disease models, including atherosclerosis, liver fibrosis, myocardial injury, cancer, diabetes, and neurodegenerative diseases, by regulating autophagy and multiple downstream signaling pathways. These compounds regulate autophagy in disease- and cell-specific manners via core pathways, including the AMPK/mTOR, PI3K/AKT, Nrf2, and SIRT1 pathways. Their modes of action include activating basal autophagy to clear damaged organelles, reducing lipid accumulation, inhibiting inflammation and fibrosis, or restoring homeostatic autophagy levels under pathological conditions, thereby alleviating cell damage and maintaining cellular homeostasis. The representative flavonoids reviewed in this article, including apigenin, luteolin, baicalein, quercetin, kaempferol, catechin, proanthocyanidins, and delphinidin, have been shown in preclinical studies to possess clear autophagy-regulating activities and pharmacological effects, showing good drug development potential and high safety.

Notably, the clinical translation of these compounds still faces several key challenges. First, most current investigations focus on cell-based and animal models in vitro, with non-uniform experimental doses, administration routes, and intervention durations, resulting in insufficient comparability and reproducibility of results. Second, flavonoids commonly suffer from poor water solubility, low membrane permeability, significant first-pass metabolism, and low oral bioavailability, leading to inadequate in vivo exposure and a notable gap between in vitro and in vivo outcomes. Furthermore, the bidirectional regulatory effects of different flavonoids on autophagy at different disease stages and their tissue specificity have not been systematically elucidated, and the dose–response relationships require in-depth investigation. Finally, existing research has mostly focused on canonical autophagy pathways, while the exploration of selective autophagy (such as mitophagy and lipophagy) and its networked regulatory mechanisms remains relatively limited.

In summary, flavonoids can exert multiple effects, including anti-inflammatory, antioxidant, anti-apoptotic, immunomodulatory, and anti-fibrotic activities by regulating autophagy, thereby ameliorating metabolic disorders, alleviating tissue damage, and delaying disease progression, demonstrating strong potential for application in the prophylaxis and therapy of metabolic, inflammatory, and degenerative diseases. Subsequent studies should additionally clarify the biochemical mechanisms and signaling pathways by which flavonoids regulate autophagy, optimize drug delivery methods and formulation technologies, and conduct high-quality, standardized clinical translational studies to facilitate the translation of flavonoids from fundamental studies to clinical applications, providing safe, efficient, and natural intervention strategies for the prophylaxis and treatment of associated diseases.

## Data Availability

Data are contained within the article.

## Abbreviations

AKT	Protein Kinase B
AMPK	AMP-Activated Protein Kinase
ATG	Autophagy-Related Gene
Bax	Bcl-2-Associated X Protein
Bcl-2	B-Cell Lymphoma 2
ECM	Extracellular Matrix
FOXO3	Forkhead Box O3
JNK	C-Jun N-Terminal Kinase
LC3	Microtubule-Associated Protein 1 Light Chain 3
mTOR	Mammalian Target of Rapamycin
NAFLD	Non-Alcoholic Fatty Liver Disease
NF-κB	Nuclear Factor Kappa-Light-Chain-Enhancer of Activated B Cells
NLRP3	NOD-Like Receptor Family Pyrin Domain Containing 3
Nrf2	Nuclear Factor Erythroid 2-Related Factor 2
OP	Osteoporosis
p62	Sequestosome-1 (SQSTM1)
PD	Parkinson’s Disease
PI3K	Phosphoinositide 3-Kinase
ROS	Reactive Oxygen Species
SCI	Spinal Cord Injury
SIRT1	Sirtuin 1
TGF-β	Transforming Growth Factor Beta
TNBC	Triple-Negative Breast Cancer
ULK1	Unc-51 Like Autophagy Activating Kinase 1
